# An unexpected ECG finding

**DOI:** 10.1007/s12471-015-0757-7

**Published:** 2015-10-08

**Authors:** M.J. Kuiper, A.R. Willems, A.A.M. Wilde

**Affiliations:** 1 Department of Internal Medicine, Sint Lucas Andreas Hospital, Amsterdam, The Netherlands; 2 Department of Cardiology, Haga Teaching Hospital, The Hague, The Netherlands; 3 Department of Cardiology, Sint Lucas Andreas Hospital, Amsterdam, The Netherlands; 4 Department of Cardiology, Academic Medical Centre, Amsterdam, The Netherlands

In this case we present a 51-year-old, female patient of Pakistani origin. Her medical history includes hepatitis C induced Child-Pugh B liver cirrhosis and she had recently been recently admitted to our hospital due to ascites with poor response to medical therapy. Currently the patient was referred to our emergency department because of nausea and abdominal discomfort. She had no cardiac medical history and a low cardiovascular risk profile. Physical examination revealed dehydration and mild diffuse abdominal pain. The patient was not experiencing fever. The laboratory results mainly showed hyperkalaemia (8.6 mmol/l). The cardiology consultant was contacted after the ECG (Fig. 1) was taken.

**Fig. 1:**
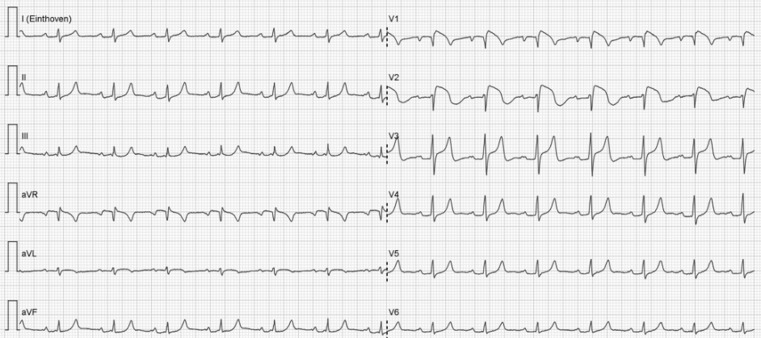
ECG at presentation

What is your diagnosis?

## Answer

You will find the answer elsewhere in this issue.

